# Renal Cell Carcinoma (RCC) Tumors Display Large Expansion of Double Positive (DP) CD4+CD8+ T Cells With Expression of Exhaustion Markers

**DOI:** 10.3389/fimmu.2018.02728

**Published:** 2018-11-26

**Authors:** Laurence C. Menard, Paul Fischer, Bijal Kakrecha, Peter S. Linsley, Erik Wambre, Maochang C. Liu, Blake J. Rust, Deborah Lee, Becky Penhallow, Nataly Manjarrez Orduno, Steven G. Nadler

**Affiliations:** ^1^Translational Medicine, Bristol-Myers Squibb, Princeton, NJ, United States; ^2^Benaroya Research Institute at Virginia Mason, Seattle, WA, United States

**Keywords:** renal cell carcinoma (RCC), CD4+CD8+ T cells, T cell dysfunction, TIM-3, PD-1, clonal expansion

## Abstract

Checkpoint inhibitors target the inhibitory receptors expressed by tumor-infiltrating T cells in order to reinvigorate an anti-tumor immune response. Therefore, understanding T cell composition and phenotype in human tumors is crucial. We analyzed by flow cytometry tumor-infiltrating lymphocytes (TILs) from two independent cohorts of patients with different cancer types, including RCC, lung, and colon cancer. In healthy donors, peripheral T cells are usually either CD4+ or CD8+ with a small percentage of CD4+ CD8+ DP cells (<5%). Compared to several other cancer types, including lung, and colorectal cancers, TILs from about a third of RCC patients showed an increased proportion of DP CD4+CD8+ T cells (>5%, reaching 30–50% of T cells in some patients). These DP T cells have an effector memory phenotype and express CD38, 4-1BB, and HLA-DR, suggesting antigen-driven expansion. In fact, TCR sequencing analysis revealed a high degree of clonality in DP T cells. Additionally, there were high levels of PD-1 and TIM-3 expression on DP T cells, which correlated with higher expression of PD-1 and TIM-3 in conventional single positive CD8 T cells from the same patients. These results suggest that DP T cells could be dysfunctional tumor-specific T cells with the potential to be reactivated by checkpoint inhibitors.

## Introduction

Checkpoint inhibitors target inhibitory receptors expressed by tumor-infiltrating T cells in order to reinvigorate an anti-tumor immune response. Despite unprecedented response rates to anti-CTLA4 and anti-PD-1/PDL1 agents in some types of advanced cancers such as melanoma or non-small cell lung cancer (1–4), responses across tumor types are highly variable (5). To effectively reject tumor cells, immunogenic antigens have to be generated (5, 6) and accessible to antigen-specific T cells, which need to infiltrate the tumor. In addition, for the therapy to be effective, there presumably have to be indications that the targeted pathway is engaged [PDL1 expression in tumor samples (4) or PD-1 expression by T cells (7) for instance]. The presence or absence of each of these prerequisites is highly variable across tumor types and patients. Even tumor-infiltrating CD8 T cells are a highly heterogeneous subset (8). Hence, a better understanding of the heterogeneity of tumor-infiltrating T cell function and phenotype across tumor types is crucial to guide therapies and tumor selection for better response rates.

Outside the thymus, CD4 and CD8 expression on T cells are usually mutually exclusive and tightly regulated by the transcription factors Runx3 and ThPOK (9, 10).Nevertheless, CD4+CD8+ double positive (DP) T cells have been described in intestinal tissue (11), in the blood and/or tissues of patients with chronic viral infections (human immunodeficiency virus, cytomegalovirus and hepatitis C virus) (12–14) and in some cancers (melanoma, breast, and metastatic colorectal cancer) (15–19). Both in the context of chronic viral infection and cancer, several studies reported an enrichment of antigen-specific cells in the DP T cell compartment suggesting that DP T cells may be important for mediating the anti-viral/tumor response.

Here, we characterize the frequencies and phenotypes of DP T cell subsets across several tumor types, and we describe the expansion of DP T cells in RCC tumors. DP T cells display an antigen-experienced phenotype with expression of activation markers and evidence of antigen-driven clonal expansion. In addition, high expression of inhibitory receptors PD-1 and TIM-3 make these patients potential good candidates for checkpoint inhibitor therapies.

## Materials and methods

### Patient cohorts and samples

Three independent cancer patient cohorts were analyzed for this study: a discovery cohort, evaluating tumor-infiltrating lymphocytes on lung cancer, colorectal cancer (CRC) and RCC tumor samples; a replication cohort focused on RCC, with 18 non-RCC tumor samples for comparison; a public dataset of densely-phenotyped RCC TILs (20). Samples for the discovery and replication cohorts were provided by Folio-Conversant Bio (AL), MT Group (CA), Rutgers-CINJ or the Benaroya Research Institute. All patients gave written informed consent at time of sample collection according to IRB protocols of each provider. Patient demographics and cancer stages are indicated in Tables 1, 2 for discovery and replication cohorts, respectively. Patients were not on checkpoint inhibitors at the time of collection, and except for one patient with CRC in the discovery cohort and one patient with a gastrointestinal stromal tumor in the replication cohort, were not receiving chemotherapy. Most tumors were stage I and II, and not metastatic. The replication cohort was focused on RCC.

**Table 1:** Patient demographics and clinical annotations for discovery cohort.

**Cancer type**	**Patients, *n***	**females, %**	**Mean age (SD)**	**Stage I (%)**	**Stage II (%)**	**Stage III (%)**	**Stage IV (%)**	**Chemotherapy and radiotherapy at collection, *n***
RCC	21	35	59 (12)	53	16	26	5	0
CRC	16	31	69 (13)	6	25	56	13	1
Lung	16	44	70 (9)	38	25	37	0	0

**Table 2:** Patient demographics and clinical annotations for replication cohort.

**Cancer type**	**Patients**, ***n*** (**with annotations)**	**females, %**	**Mean age (SD)**	**Stage I (%)**	**Stage II (%)**	**Stage III (%)**	**Stage IV (%)**	**Chemotherapy at collection, *n***
RCC	23 (21)	21	60 (13.5)	71	24	5	0	0
Others	18 (14)	82	64 (14)	42	21	7	0	1

Tumor samples and matching blood were received and processed within 24 h of collection. To isolate tumor-infiltrating lymphocytes (TILs), tumors were dissociated either with the Tumor Dissociation Kit (Miltenyi Biotec) for the discovery cohort or with a mild cocktail of collagenase I (50 U/ml), II (18 U/ml), IV (52 U/ml) and elastase (0.1 U/ml)(Worthington Biochemical) for the replication cohort.

### Flow cytometry

For the discovery cohort, ACD-whole blood pre-treated with RBC lysis buffer (Biolegend) and freshly isolated TILs were washed with 1X PBS and stained for viability with LIVE/DEAD Fixable Near-IR Dead Cell kit (Thermo Fisher Scientific). Non-specific binding was prevented by blocking Fc receptors with Human AB serum (Gem Cell). Cells were stained with an antibody cocktail that contained CD3-BUV395(SK7, BD), CD4-BV785 (OKT4, Biolegend), CD8a-BV605 (SK1, BD), CD45-AF700 (HI30, BD).

For the replication cohort, heparin whole blood pre-treated with red blood cells sedimentation buffer (Miltenyi) and freshly isolated TILs were washed with 1X PBS and stained for viability with LIVE/DEAD Fixable Near-IR Dead Cell dye (Thermo Fisher Scientific). Non-specific binding was prevented by blocking Fc receptors with Human Gamma Globulin (Jackson ImmunoResearch). Cells were stained with an antibody cocktail that contained CD45-BV480 (HI30, BD), CD3-BUV496 (UCHT1, BD), CD4-AF700 (SK3, Biolegend), CD8-BUV395 (RPA-T8, BD), CD45RO-BV421 (UCHL1, Biolegend), CCR7-BV711 (G043H7, Biolegend), PD-1-APC (MIH4, BD), GITR-BV785 (6G10–BMS), TIGIT-PE (MBSA43, eBioscience), ICOS-PE-Cy7 (C398.4A, Biolegend), TIM-3-BB515 (7D3, BD) and OX40-BV605 (ACT35, Biolegend). When enough TIL were recovered, a second panel containing CD45-BV480 (HI30, BD), CD3-BUV496 (UCHT1, BD), CD4-AF700 (SK3, Biolegend), CD8-BUV395 (RPA-T8, BD), CD45RO-BV421 (UCHL1, Biolegend), CCR7-BV711 (G043H7, Biolegend), PD-1-APC (MIH4, BD), and CD38-PE-Cy7 (HB7, Biolegend) was also used. Stained cells were then washed and fixed with 1x BD lyse buffer (BD Biosciences). For both cohorts, samples were run on a LSR Fortessa (BD). Data analysis was performed with FlowJo or Cytobank.

### Spanning-tree progression analysis of density-normalized events (SPADE)

The SPADE algorithm (21) was run in Cytobank on single viable CD45+CD3+ gated cells from 10 RCC samples with more than 5% DP T cells from the replication cohort, using surface markers (CD4, CD8, TIGIT, ICOS, TIM-3, PD-1, CD45RO, CCR7, OX40, GITR). The down-sampled event target was 10% and the target number of nodes was 100.

### Analysis of mass cytometry dataset

Publicly available data from RCC TIL were uploaded from https://premium.cytobank.org/cytobank/experiments/101725 into Cytobank for gating and analysis.

### Single cell sort and TCR analysis

Single cells were sorted directly into SMARTseq v4 (Takara) lysis buffer to release RNA. Reverse transcription was performed followed by PCR amplification to generate full length amplified cDNA. Sequencing libraries were constructed using a modified protocol of the NexteraXT DNA sample preparation kit (Illumina) to generate Illumina-compatible barcoded libraries. Libraries were pooled and quantified using a Qubit® Fluorometer (Life Technologies). Dual-index, single-read sequencing of the pooled libraries was carried out on a HiSeq2500 sequencer (Illumina) with 58-base reads, using HiSeq v4 Cluster and SBS kits (Illumina) with a target depth of 1 million reads per sample. FASTQs were aligned to a human reference genome to generate gene counts.

### Statistics

Statistical analyses were performed with GraphPad Prism 7. For comparisons between subsets from the same samples, the Wilcoxon test was used. *P* values were adjusted for 3 comparisons. Correlations between continuous variables were analyzed with Spearman. A *P-*value of 0.05 or lower was considered significant.

To compare the degree of clonal expansion between different groups with different numbers of libraries/cells, we repetitively down-sampled (100x) to a reduced, standard sample size (*N*~25 cells), and calculated the mean fraction of enrichment for each cell subset from each donor in the down-samplings. Differences between the mean fractions of expanded TCRs for the different subsets were evaluated with a two-sided *t*-test. Statistical differences in TCR junction sharing between subsets were evaluated with a Fischer exact test.

## Results

### RCC tumors display a large expansion of CD4+CD8+ double positive T cells

**Figure 1:**
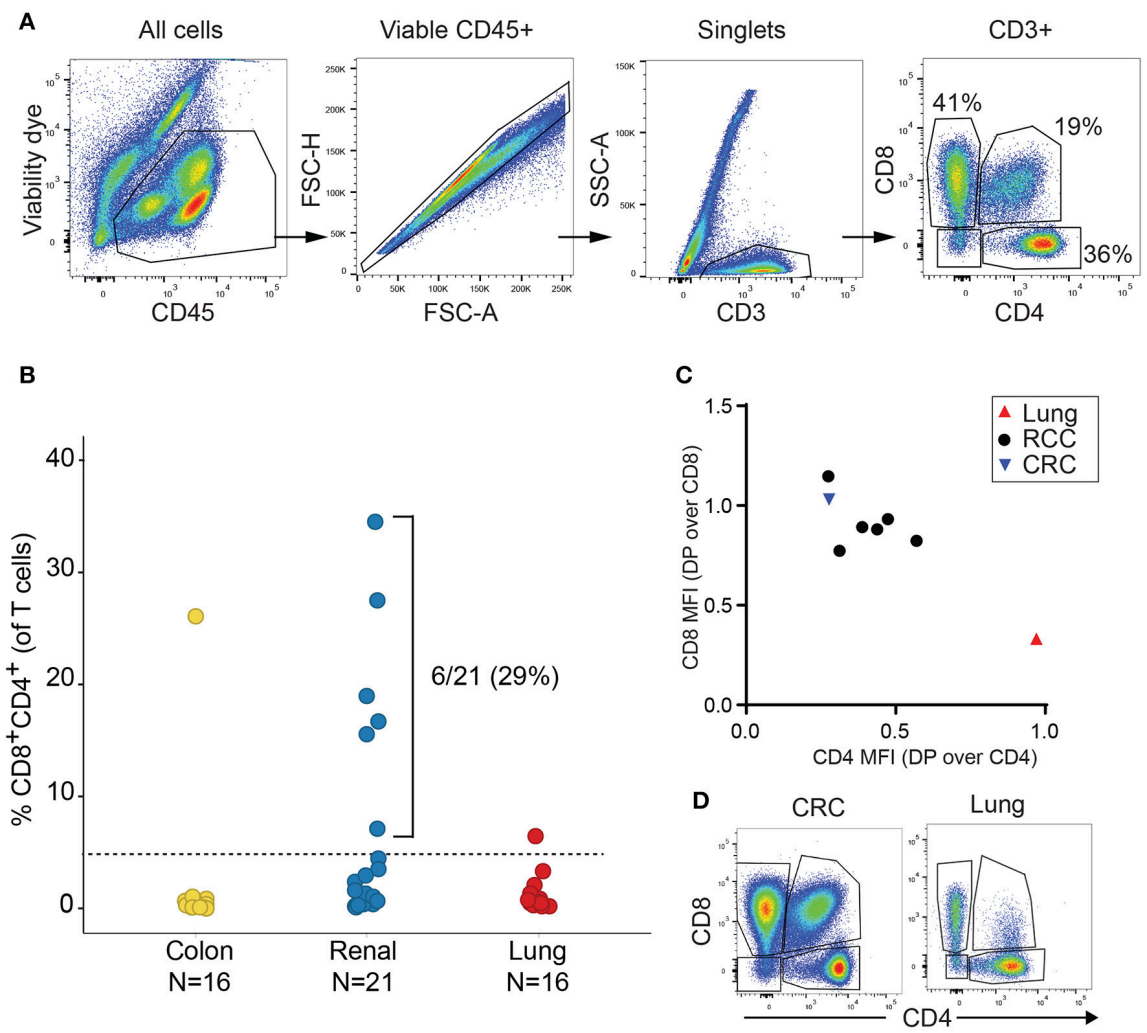
Expansion of DP T cells in RCC (discovery cohort). **(A)** Example of gating strategy for flow cytometry analysis of a RCC sample: CD4 and CD8 expressions were visualized after gating on viable CD45+ single CD3+ T cells. **(B)** Frequencies of DP CD4+CD8+ T cells in TILs from a set of CRC, RCC and lung tumors. Horizontal line is the 5% cut-off. **(C)** Plot representing ratio of CD4 MFI in DP over CD4+CD8- T cells on x-axis and of CD8 MFI in DP over CD4-CD8+ on y-axis. Each dot represents a sample with > 5% DP T cells as identified in Figure 1B. **(D)** Dot plots of CD4 vs. CD8 expression in the CD3+ gate of the CRC and the lung tumor samples with > 5% DP.

We initially analyzed the proportion of TIL T cell subsets based on CD4 and CD8 expression in a discovery cohort of 21 RCC, 16 lung cancer and 16 CRC samples. Cell doublets were carefully excluded as shown on the gating strategy in Figure 1A. Although CD4+CD8+ DP T cells represented less than 2% of T cells in a majority of tumor samples, frequencies of CD4+CD8+ DP T cells above 5% of T cells were observed in 6/21 of RCC samples, 1/16 lung cancer and 1/16 CRC samples (Figure 1B). CD4 and CD8 median fluorescence intensity (MFI) in DP T cells were normalized to CD4 and CD8 MFI of conventional CD4 and CD8 T cells from the same samples, respectively, for comparison. All RCC and CRC DP T cells had a CD8 expression level close to conventional CD8+CD4- T cells (ratio of CD8 MFI in DP over CD8 MFI in CD8: 0.77–1.1) (Figure 1C). CD4 was expressed at lower level on DP T cells than on CD4+CD8- T cells (ratio of CD4 MFI in DP over CD4 MFI in CD4: 0.28–0.57). The expanded DP T cell subset observed in the lung cancer patient (6.5% of T cells) showed a different profile with CD4 expression similar to CD4 T cells (ratio of CD4 MFI in DP over CD4 MFI in CD4: 0.97) and significantly lower CD8 expression than CD8 T cells (ratio of CD8 MFI in DP over CD8 MFI in CD8 cells: 0.33) (Figures 1C,D). Thus, the DP T cells observed in the 6 RCC and 1 CRC samples were CD8+CD4^low^, whereas the DP T cells from the lung sample were CD4+CD8^low^ and likely constitute a distinct subset of DP T cells (13, 22).

**Figure 2:**
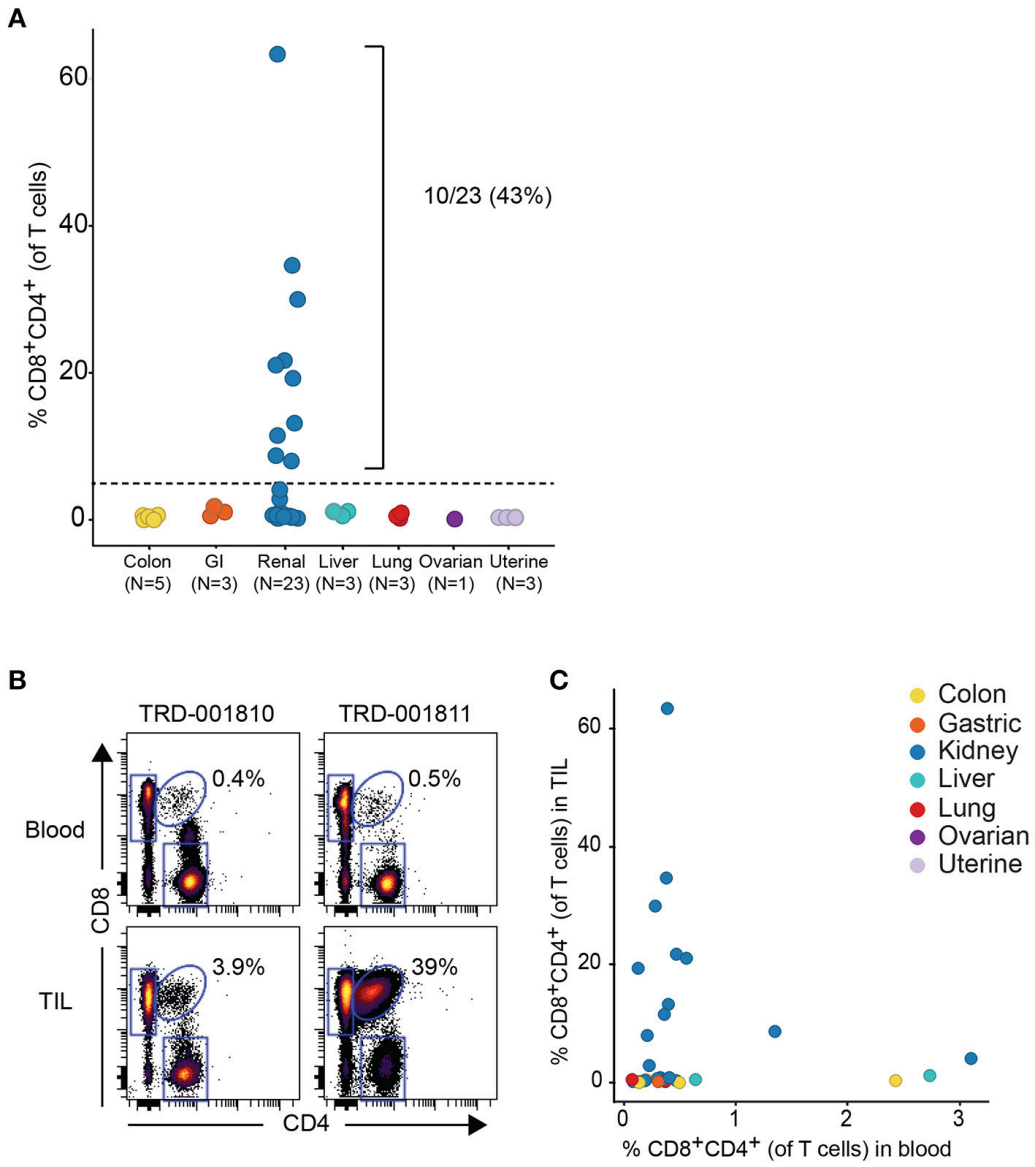
Expansion of DP T cells in RCC (replication cohort)**. (A)** Summarized frequencies of DP T cells in a replication RCC-enriched cohort that included TIL samples from 23 RCC vs. 18 non-RCC tumors. The dotted line at 5% is the arbitrary cutoff used to determine expansion of DP T cells. **(B)** Representative example of dot plots for CD4 and CD8 expression, gated on single viable CD45+CD3+ T cells, from 2 RCC TILs and matching blood samples. The elliptical gate shows the presence of CD4+CD8+ DP T cells. **(C)** Frequencies of DP T cells in blood (x-axis) vs. TILs (y-axis).

Given that the expanded DP T cell population occurred more commonly in RCC vs. other tumor types, we obtained a replication cohort focused on RCC tumors that we compared to 18 diverse, non-RCC tumor samples. Consistent with data from the discovery cohort, frequencies of DP T cells above 5% of T cells were also observed in 10/23 RCC samples, reaching up to 62% of T cells in a sample, with no DP T cell expansion in the other tumor types (Figure 2A). By contrast, analysis of matching blood did not reveal higher frequencies of circulating DP T cells in the blood of patients with expanded tumor DP T cells, suggesting that these DP T cells may not recirculate (Figures 2B,C).

Analysis of TIL T-cell subsets across several tumor types in 2 independent sample sets revealed an expansion of CD4+CD8+ T-cell subset enriched in RCC. We thus went on to characterize further DP T cells in the replication cohort.

### No correlation between DP expansion and tumor stage

Histology and pathology data in the replication cohort were available for 18 out of 23 samples. Samples with more than 5% DP T cells were all of the clear cell RCC type. Stage and grade were similar between patients with and without expanded DP T cells. Age and tumor size did not correlate either with the expansion of DP T cells (Table 3). We were thus unable to correlate the expansion of DP T cells with any of the available histology and pathology data. In addition, RCC samples in both the discovery and replication cohorts were all treatment naive, which rules out a potential effect of treatment on the expansion of DP T cells (Tables 1, 2).

**Table 3:** Demographics and clinical annotations for 18 RCC patients with more or less than 5% DP T cells from the replication cohort.

**Parameter**	**DP<5% (*n* = 9)**	**DP>5% (*n* = 9)**
Mean age (SD)	61 (14)	58 (13)
Female, *n* (%)	1 (11)	3 (33)
Stage, *n*		
I	6	7
II	2	2
III	1	0
Grade, *n*		
1	1	0
2	4	6
2–3	1	2
3	2	1
4	1	0
Histology: clear cell vs. others, *n* (%)	5 (56)	9 (100)
Mean tumor size (SD)	7.6 (6.2)	5.6 (1.9)

### Double positive T cells are a subset of CD8 T cells with an effector memory phenotype

**Figure 3:**
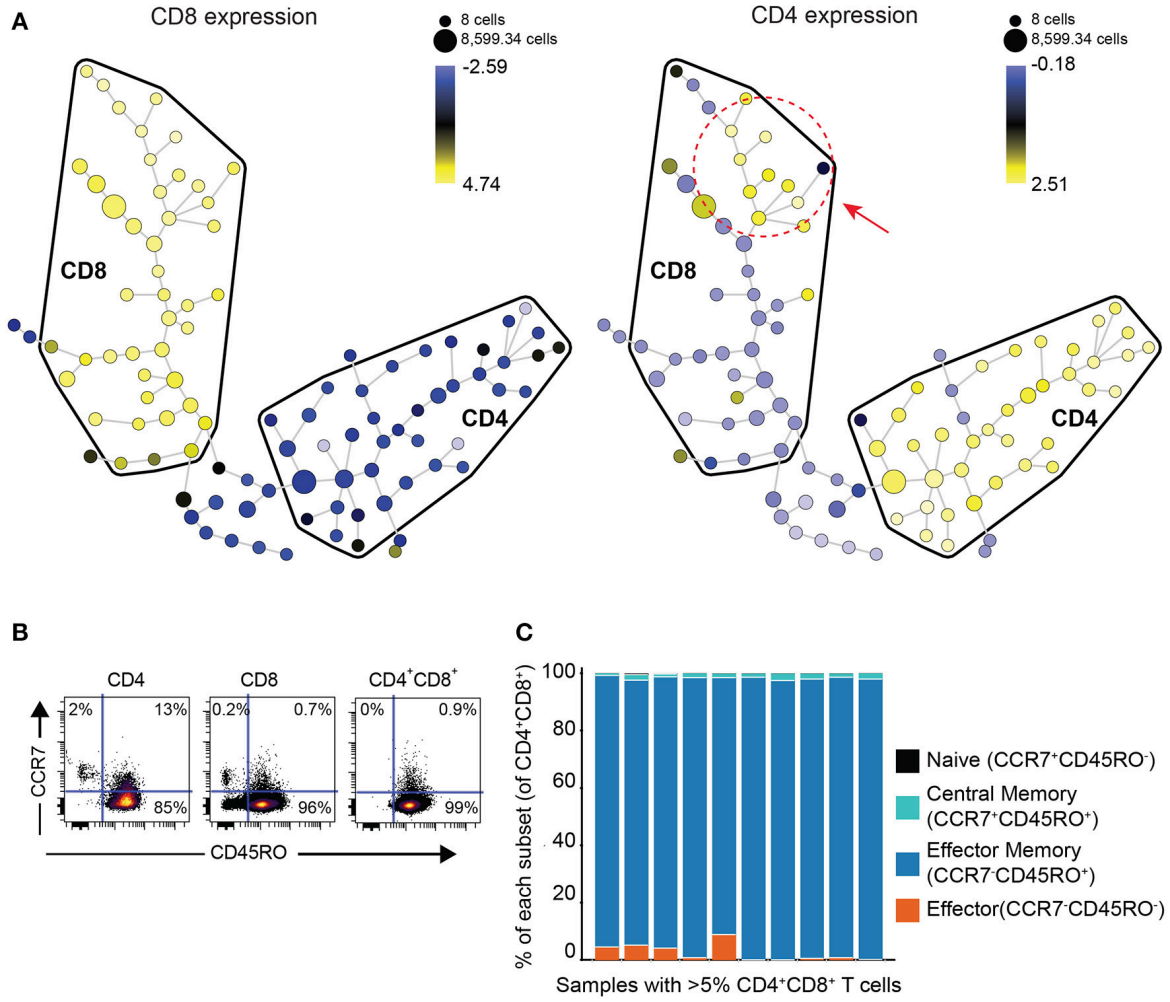
DP T cells appear to be a subset of CD8+ T cells**. (A)** Representative example of a SPADE-tree generated with surface markers (CD4, CD8, TIGIT, ICOS, TIM-3, PD-1, CD45RO, OX40, GITR, CCR7) on the replication cohort in the 10 samples with >5% DP T cells (red circle and arrow indicate zone where DP T cells cluster). The size of the nodes represents the number of cells, and the color represents the transformed median expression level of CD8 (left panel) or CD4 (right panel). **(B)** Representative dot plot of CD45RO (x-axis) and CCR7 (y-axis) in CD4+, CD8+, and DP gated T cells. **(C)** Summary of cell frequencies in each quadrant shown in **(B)** for DP T cells from RCC samples with >5% DP cells (replication cohort).

In order to determine if DP T cells are either an independent subset of T cells or a subset of CD8 T cells that upregulated CD4 or vice versa, we performed clustering analysis of the phenotype of DP T cells with the Spanning-tree Progression Analysis of Density-normalized Events (SPADE) algorithm using surface markers (CD4, CD8, TIGIT, ICOS, TIM-3, PD-1, CD45RO, CCR7, OX40, GITR) in 10 samples of the replication cohort with more than 5% DP T cells (Figure 2A). CD4+ and CD8+ T cells mostly clustered in different areas or “bubbles” according to CD8 and CD4 expression. Interestingly, DP T cells clustered with conventional CD4–CD8+ T cells as revealed by CD4 expression within the CD8 bubble, suggesting that they are a subset of CD8+ T cells (Figure 3A, Supplementary Figure 1). Expression of CD4 and CD8 is under the tight regulation of transcription factors that prevent co-expression, except in thymic cells during early development. Analysis of CD45RO and CCR7 expression showed that DP T cells were mostly CD45RO+CCR7-, characteristic of an effector memory phenotype, meaning that DP T cells are antigen experienced and unlikely to be recently egressed thymic DP T cells (Figures 3B,C).

### Double positive T cells express activation markers

**Figure 4:**
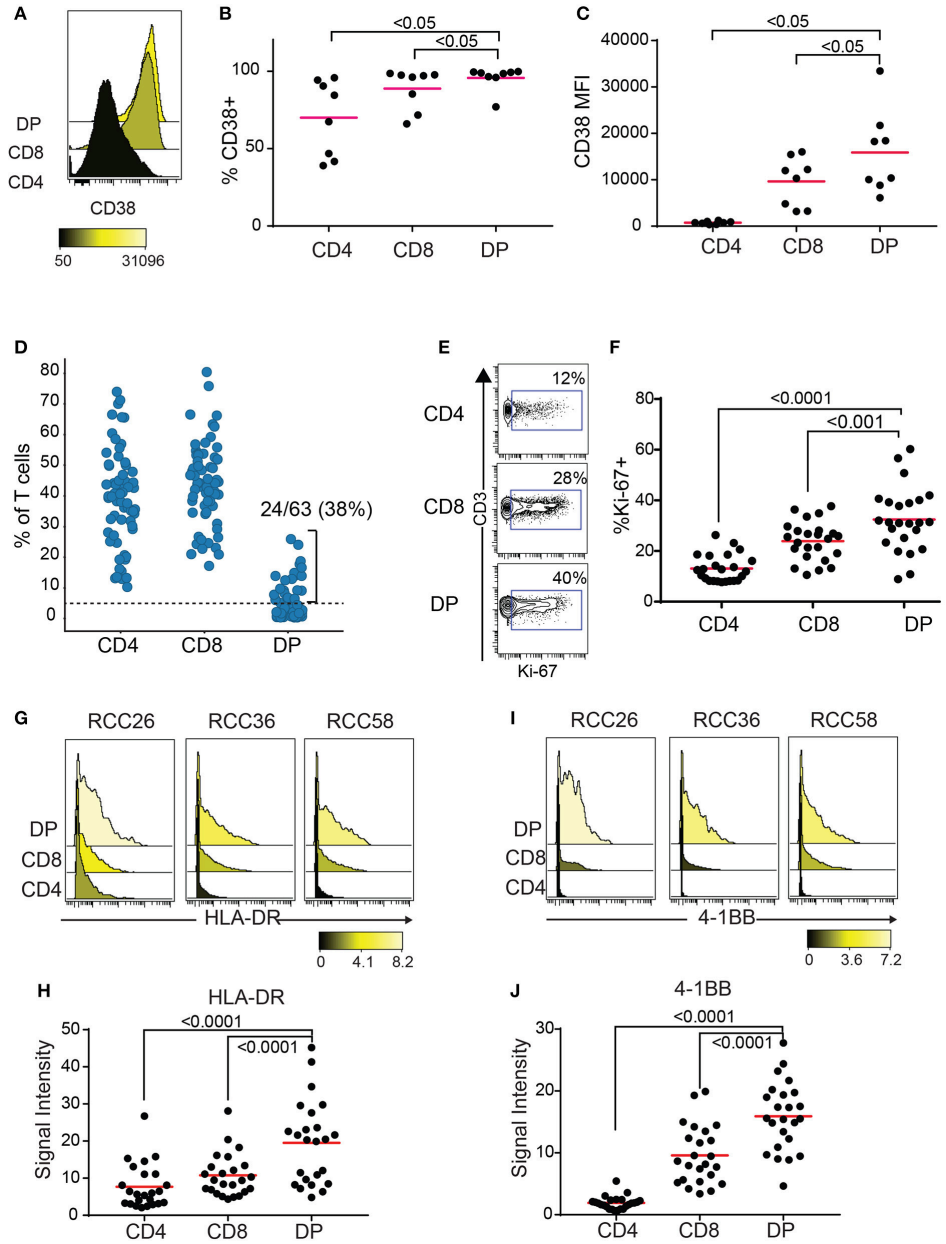
DP T cells show markers of activation. **(A–C)** CD38 expression by CD4, CD8 and DP T cells in 8/10 samples from replication cohort with >5% DP T cells. **(A)** Representative histogram of CD38 expression in the 3 subsets of T cells. The color bar represents the median fluorescence intensity (MFI). **(B)** Frequencies of CD38+ and **(C)** CD38 MFI in CD38+ cells for the 3 subsets for samples with > 5% DP. **(D)** Frequencies of CD4+, CD8+, and DP T cells (of TIL CD3+ T cells) in a publicly available RCC dataset (20). Only samples with >1,000 CD3+ T cells were used (*N* = 63). The dotted line at 5% is the cutoff used to determine expansion of DP T cells. **(E)** Representative density plot for Ki-67 for CD4, CD8 and DP T cells. The gate represents the Ki-67+ cells and the number is the frequencies of cells in the Ki-67+ gate. **(F)** Summary of %Ki-67+ cells in CD4, CD8 and DP T cells for samples with more than 5% DP. **(G)** Representative histogram of HLA-DR expression for CD4, CD8, and DP T cells from 3 samples. The color bar represents the signal intensity. **(H)** Summary of HLA-DR signal intensity in CD4, CD8 and DP T cells. **(I)** Representative histogram of 4-1BB expression for CD4, CD8 and DP T cells from 3 samples. The color bar represents the signal intensity. **(J)** Summary of 4-1BB signal intensity in CD4, CD8, and DP T cells. For **F–J**, only samples with >5% DP cells were used. Adjusted p values are indicated on plots (Wilcoxon test).

CD38 is an activation marker upregulated by inflammatory cytokines (23) and potentially enriched in antigen-experienced T cells (24, 25). Blood HLA-DR+CD38+ CD8 T cells show expansion of tumor-infiltrating clones following anti-PD1 treatment (26). Enough TIL were recovered from 8 out of the 10 RCC samples with expanded DP T cells from the replication cohort to run a second flow cytometry panel to measure CD38 expression. Both frequencies of CD38+ cells and median fluorescence intensity (MFI) were highest in DP T cells, followed by CD8, then CD4 T cells (Figures 4A–C). In order to gain more information on the activation status of DP T cells, we analyzed a publicly available mass cytometry dataset that contains data on expression of additional activation markers HLA-DR, 4-1BB, and Ki-67 in RCC T cell subsets (20). In this dataset, 24/63(38%) RCC samples showed more than 5% of CD4+/loCD8+ DP T cells, confirming the expansion of this subset in RCC (Figure 4D, Supplementary Figure 2). Similarly to the SPADE analysis performed on the flow cytometry expression data generated on the replication cohort, SPADE trees generated on this mass cytometry dataset showed clustering of DP T cells within the CD8 “bubble” (Supplementary Figure 3). The frequency of Ki-67+ cells was higher in DP T cells than in CD8 T cells, and in CD8 T cells compared to CD4 T cells (Figures 4E,F). HLA-DR and 4-1BB (27), markers of activation believed to be enriched for antigen-specific T cells, were also expressed at higher levels by DP T cells compared to conventional CD8 and CD4 T cells (Figures 4G–J).

### Double positive T cells show high level of clonal expansion

**Figure 5:**
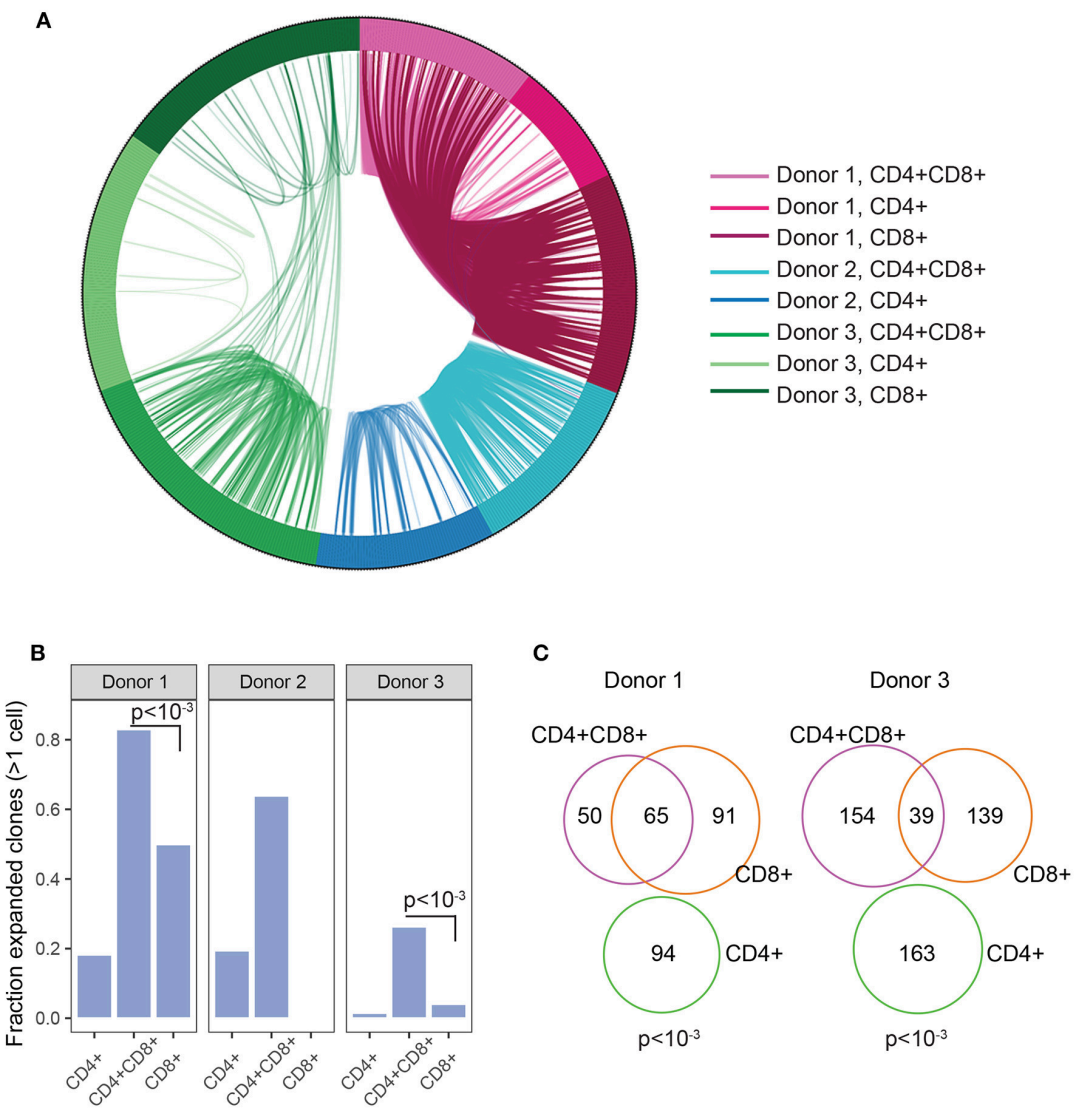
Clonal expansion in DP T cells**. (A)** Plot showing TCR sharing across CD4, CD8, and DP subsets from 3 donors. Each segment of the circle represents a cell with a rearranged TCR sequence and identical TCR rearrangements are connected with an arc. **(B)** Fraction of expanded clones across CD4, CD8, and CD4+CD8+ DP T subsets and donors. Clonal expansion was significantly higher in DP than in CD8 T cells in donor1 and donor3 (*p* < 10^−3^, indicated on plots) **(C)** Venn diagram representing TCR junction sharing across CD4+ (green circle), CD8+ (orange circle) and CD4+CD8+DP (purple circle) T cell subsets for donor 1 and donor 3. Numbers represent the number of TCR junctions analyzed. Fischer's p values are indicated on plot.

The enrichment of DP TIL T cells with an activated/antigen experienced phenotype suggests antigen-driven expansion of T cells. To confirm this hypothesis, TCR clonal analysis was performed. An antigen-driven expansion should lead to an increase in the frequency of specific clones and a diminished diversity. Single CD4+, CD8+, and DP T cells were sorted from 3 RCC TIL samples. Libraries were obtained for single CD4, CD8, and DP T cells from 2 donors and for single CD4 and DP T cells from the third donor. TCRα and TCRβ chains sequencing analysis revealed that in each of the 3 donors, many TCR pairs were shared between DP T cells, showing relatively higher clonal expansion compared to CD4 and CD8 T cell subsets and suggesting antigen-driven expansion of DP T cells. By contrast, the CD4 T cell subset showed limited clonal expansion (Figures 5A,B). Analysis of CD8+ T cells in 2 of the 3 donors also revealed that many clones are shared between CD8+ and DP T cells, consistent with the hypothesis that they are the same or closely related subsets (Figure 5C). *CD8A* and *CD8B* were co-expressed in single DP and CD8 T cells, indicating that CD8α/CD8β heterodimers are likely expressed in both subsets (Supplementary Figure 4).

### Double positive T cells express inhibitory receptors

**Figure 6:**
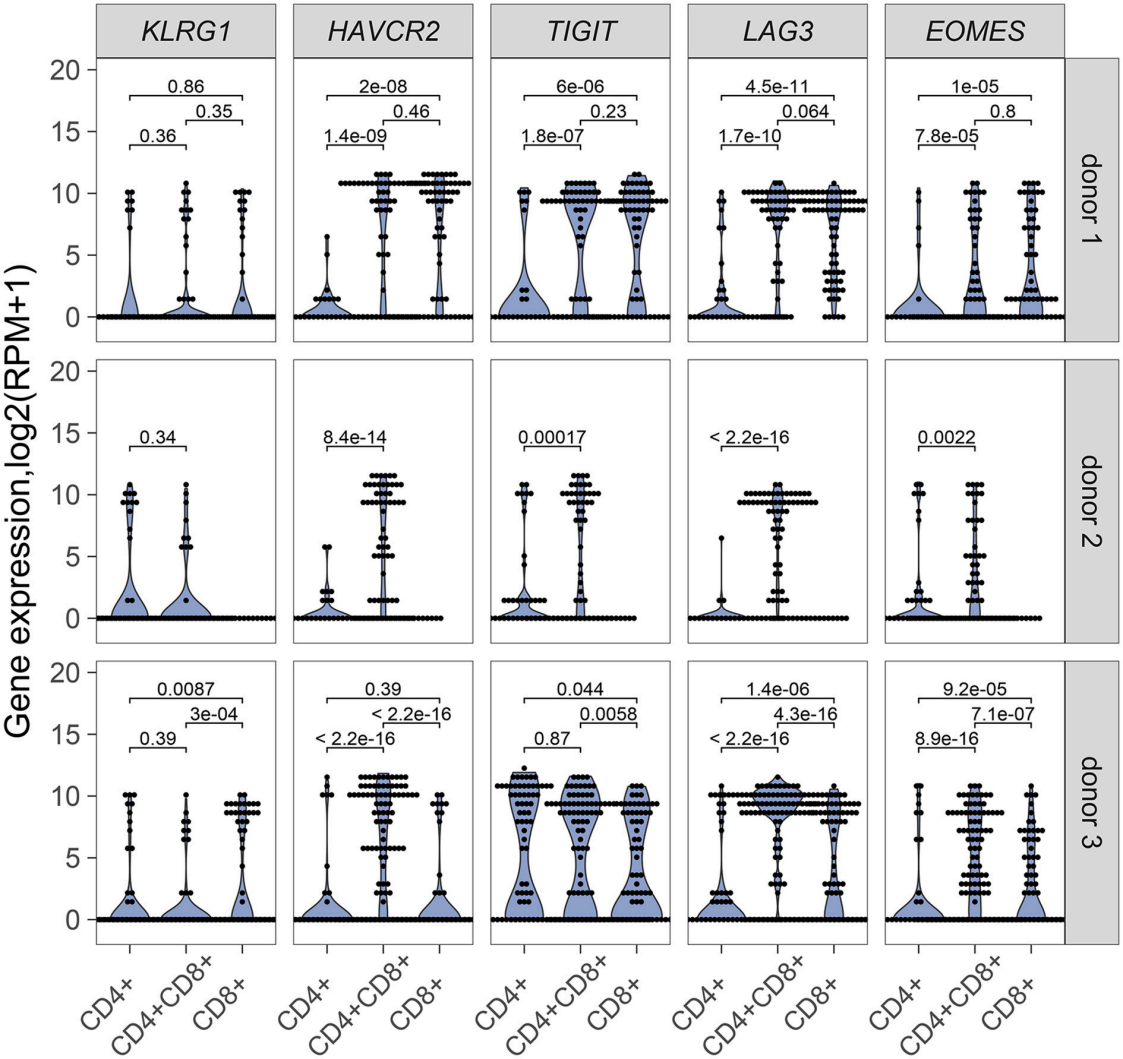
mRNA expression of inhibitory receptor genes *KLRG1, HAVRC2, TIGIT, LAG3* and transcription factor gene *EOMES* in single CD4+, CD8+ and CD4+CD8+ DP T cells from donor 1 and donor 3 and in single CD4+ and CD4+CD8+ DP T cells from donor 2. Unpaired Wilcoxon test was used, p values are indicated on plots.

Our results point to an antigen-driven expansion of DP and CD8 T cells in some RCC tumors, and yet, those tumors are not rejected, suggesting strong inhibitory mechanisms. Expression of inhibitory receptors on DP T cells was therefore examined. *HAVR2*, which codes for TIM-3*, TIGIT*, and *LAG3* were generally expressed in single DP T cells from the 3 donors analyzed for TCR sequence analysis, at higher levels than single CD4 T cells and higher or similar levels as single CD8 T cells (Figure 6). *EOMES*, a key transcription factor in the regulation of CD8 T cell effector function (28), was also expressed at higher level in DP T cells than in CD4 T cells (Figure 6). DP T cells expressed *ITGAE* (codes for CD103), and *CD69*, but not *SELL, KLF2*, and *S1PR1*, suggesting a tumor resident phenotype (29, 30) (Supplementary Figure 5).

**Figure 7:**
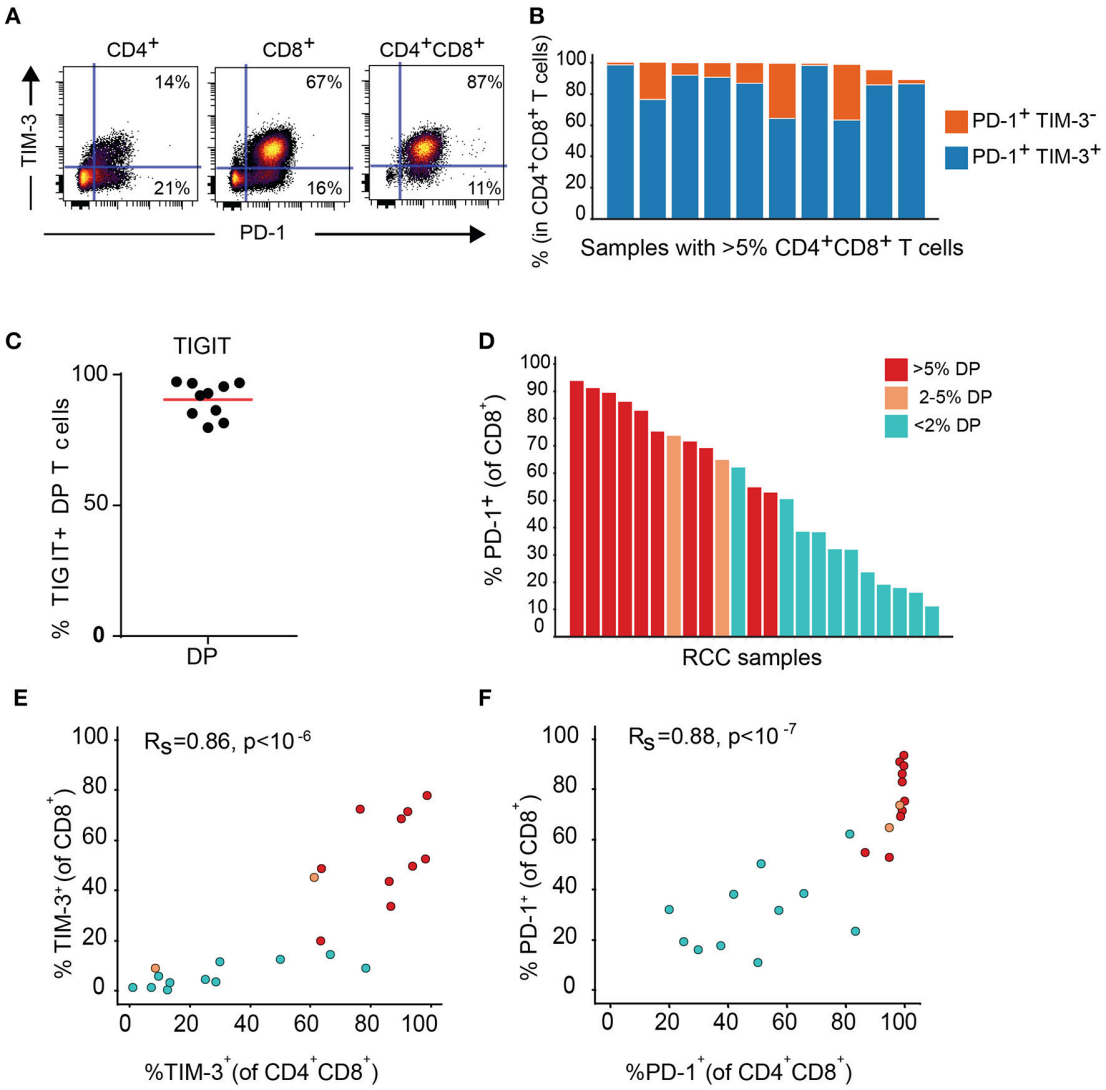
DP T cells co-express inhibitory receptors PD-1 and TIM-3**. (A)** Representative dot plot of PD-1 (x-axis) and TIM-3 (y-axis) in CD4+, CD8+, and DP T cells. **(B)** Summary of PD-1+ TIM-3+ and PD-1+ TIM-3- DP T-cell frequencies for RCC samples with >5% DP cells. **(C)** Frequencies of TIGIT+ CD4+CD8+ DP T cells. **(D)** Frequencies of PD-1+ CD8+ T cells in RCC samples with >5% DP T cells (red), 2–5% DP T cells (orange) and <2% DP T cells (aqua). **(E,F)** Correlation of TIM-3 **(E)** and PD-1 **(F)** expressions on CD4+CD8+DP and CD8+ T cells. Rs and *P-*value for Spearman correlations are shown on graphs.

Expression of TIM-3 and TIGIT, as well as PD-1, another inhibitory receptor was analyzed by flow cytometry on T cell subsets from the DP T cells in the replication cohort. Close to 100% of DP T cells in almost all donors expressed PD-1. In addition, PD-1 was highly co-expressed with TIM-3 (Figures 7A,B). TIGIT was also expressed at high level in the DP subset (Figure 7C). In addition, conventional CD8+ T cells from samples with expanded DP T cells also showed high expression of PD-1 and TIM-3 (Figures 7D–F).

## Discussion

Here we describe the expansion of CD4+CD8+ DP T cells in RCC (29–43% of samples) in three independent data sets. The other tested tumor indications, mainly lung and CRC, did not show a significant increase in DP T cells. DP T cells appeared to be a subset of clonally expanded CD8+ T cells that expressed both activation markers (HLA-DR, CD38, 4-1BB, CD137, Ki-67) and inhibitory markers (PD-1, TIM-3, and TIGIT).

Clustering analyses of flow cytometry data suggest that DP T cells are a subset of CD8 T cells that upregulate CD4. CD4 can be re-expressed upon TCR stimulation (31) and DP T cells are expanded in the blood of patients chronically infected with viruses and enriched in virus-specific T cells (12–14). The meaning of CD4 upregulation on CD8 T cells is unknown. CD4 expression on CD8 T cells was shown to enhance cytotoxic anti-viral responses (12). Decreased CD4 help may favor CD8 T cell exhaustion (32) therefore CD4 upregulation could be a way to reverse CD8 T cell exhaustion. We could speculate that CD4 expression on DP T cells might allow them to respond to MHCII antigens in an effort to overcome resistance mediated by MHCI loss observed in many tumor types (33). It would then be interesting to evaluate an association between MHCI loss and expansion of DP T cells in RCC. Could DP T cell then have a helper function? CD40L, which was upregulated on lines derived from DP T cells from melanoma samples, provided helper T function to DP T cells, leading to activation of B cells and increase in DC cross-presentation functions (18). Upregulated CD4 and CD40L on CD8 could thus perhaps be a mechanism for CD8 to counterbalance the negative effect of the tumor micro-environment on CD8 activation. A deeper understanding of this peculiar subset of CD8 T cells will likely uncover new tumor T cell biology.

Why were we able to identify these cells only in RCC? Previously published reports have described expansion of DP T cells in breast pleural effusions (15), melanoma (16), and metastatic CRC (17). We did not have any melanoma or breast tumor samples to confirm these findings, however, the frequencies of DP T cells described in those tumor types were generally within 10% of T cells, which is a significantly lower level of expansion than what observed in RCC, where DP cells could constitute more than 60% of T cells. Besides RCC, only one CRC sample in the discovery cohort showed expansion of DP T cells (26% of T cells) with CD4 and CD8 expression profiles similar to RCC DP T cells. This donor had intact mismatch repair protein expression and was likely micro-satellite stable. It was a stage III invasive adenocarcinoma. Additional stage III and metastatic stage IV CRC cases showed 1% or less of DP T cells. Therefore, we could not correlate the presence of these cells with metastasis as previously reported (17). In our RCC cohorts, patients with expanded DP T cells were essentially of the clear cell subtype without correlation with stage or grade.

The activated phenotype of DP T cells, taken together with the higher TCR repertoire clonality, suggest an antigen-driven expansion of DP T cells. A previous report had shown oligoclonal expansion of tumor-reactive effector memory T cells in TILs from two clear cell RCC patients, although whether these cells were CD4, CD8 or DP T cells was not specified (34). RCC was found to have a higher proportion of coding frameshift mutations in a pan-cancer study, potentially leading to large amounts of neoantigens and associated with a cytotoxic and CD8 signature (35). However, according to this study, lung adenocarcinoma was also a tumor type with potentially even higher frameshift neoantigens per tumor but we did not observe DP T cell expansion in lung adenocarcinoma. In addition, a more recent report did not confirm the positive association between frameshift mutations and T cell signatures in a large RCC cohort (36). Could another antigen specific to RCC explain the DP T cell expansion? Several cancer types have been associated with viral infection (head and neck or cervical cancers with human papillomavirus (HPV) or hepatic cancer with hepatitis B or C viruses, for instance) (37) although evidence for a role of oncogenic viruses in clear cell RCC is limited. Only a few studies have reported a possible link between renal cancer and HPV, hepatitis C virus, or Epstein-Barr virus infections (38) although no viral sequences were found in TCGA data from RCC samples in a recent study (37). The human genome contains a significant amount (~4.7%) of DNA from endogenous retroviruses (ERV) (39). While generally repressed in healthy tissue, expression of ERV genes can be increased in some tumors. In fact, envelop proteins from the human ERV E (HERV-E) can be re-expressed in VHL-deficient clear cell RCC tumor cells potentially triggering a T cell response and tumor rejection (40–42). Whether the antigens driving clonal expansion of DP T cells in this subset of RCC tumors are neoantigens or derived from ERV will have to be determined.

In addition to the expression of activation markers, DP T cells also express the inhibitory receptors PD-1 and TIM-3, which have been previously associated to T cell dysfunction (43, 44). PD-1 and TIM-3 were also more highly expressed by conventional CD8 T cells from patients with expanded DP T cells. These markers are upregulated following T cell activation, which is consistent with the rest of the phenotype of DP T cells. Higher Ki-67 staining in DP T cells suggests recent proliferation as Ki-67 staining will increase after 1 day and stay high for 3–6 days in the presence of TCR stimulation (45, 46), following similar kinetics of expression as TIM-3 and PD-1. In chronically HIV infected patients, a majority of circulating Ki-67+ CD4 T cells were not actively proliferating but arrested in G1 phase of the cell cycle (47). In addition, Ki-67+ T cells expressing TIM-3 were enriched in HIV-1 infected patients. TIM-3+ Ki-67+ T cells appeared dysfunctional and did not proliferate or expressed higher IFNγ upon *in vitro* re-stimulation (43). Consistent with these findings, compared to their TIM-3- counterparts, PD-1+TIM-3+ CD8 T cells from RCC tumors were shown to respond poorly to anti-CD3 and anti-CD28 stimulation as measured by IFNγ secretion. In addition, the presence of this PD-1+TIM-3+ CD8 subset was a marker of worse prognosis in RCC patients (48). Blockade of TIM-3 and PD-1 can reverse T cell dysfunction (49, 50) and high expression of PD-1 in TIL predicts response to PD-1 blockade (7). Although this hypothesis needs to be tested with functional assays, according to this published literature, high expression of PD-1 and TIM-3 on DP T cells may inhibit their function, which could be restored by blockade of inhibitory receptors. In Type 1 diabetes patients treated with teplizumab, responders showed a population of CD8 memory T cells that expressed EOMES and inhibitory receptors and retained some effector function as shown by *IFNG* and *GZB* expression, like the DP T cells in this study (51).

In conclusion, these results suggest that DP T cells might potentially be a subset of dysfunctional tumor antigen-specific CD8+ T cells with the potential to be reactivated by treatment with checkpoint inhibitors. A CD4/CD8 duplex immunohistochemistry assay might enable us to predict response to checkpoint inhibitors in RCC in the future.

## Ethics statement

This study was carried out in accordance with the recommendations of each provider's Institutional Review Board (IRB) (providers were BRI, Folio-Conversant, MT Group, CINJ) with written informed consent from all subjects. All subjects gave written informed consent in accordance with the Declaration of Helsinki. The protocol was approved by the IRB.

## Author contributions

LM, PF, PL, EW, BP, NM, and SN planned and designed experiments. PF, BK, EW, ML, BR, and BP performed experiments. LM, PF, BK, PL, EW, BP, and DL analyzed results. LM wrote the manuscript. PL, EW, NM, and SN provided intellectual input and helped preparing the manuscript.

### Conflict of interest statement

LM, PF, BK, DL, BP, NM, SN are BMS employees. PL and EW received funding from BMS. The remaining authors declare that the research was conducted in the absence of any commercial or financial relationships that could be construed as a potential conflict of interest.
