# Supplementary material for: Renal Cell Carcinoma (RCC) Tumors Display Large Expansion of Double Positive (DP) CD4+CD8+ T Cells With Expression of Exhaustion Markers
Source: Front Immunol. 2018 Nov 26;9:2728. doi: 10.3389/fimmu.2018.02728 (PMC6275222; doi:10.3389/fimmu.2018.02728)
Supplement: Supplementary file 1 [file Presentation_1.PPTX]

## Slide 1
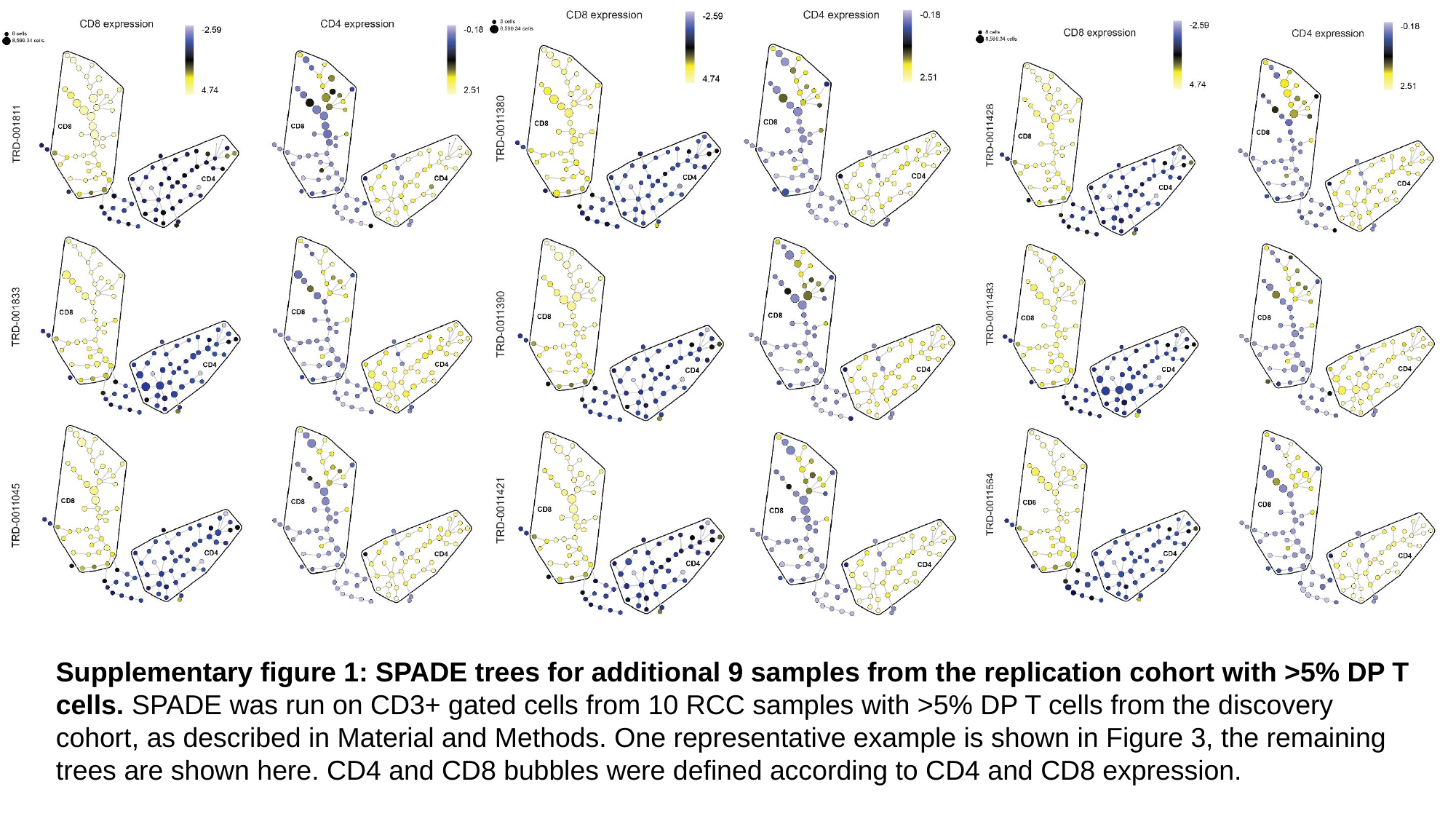

#
Supplementary figure 1: SPADE trees for additional 9 samples from the replication cohort with >5% DP T cells. SPADE was run on CD3+ gated cells from 10 RCC samples with >5% DP T cells from the discovery cohort, as described in Material and Methods. One representative example is shown in Figure 3, the remaining trees are shown here. CD4 and CD8 bubbles were defined according to CD4 and CD8 expression.

## Slide 2
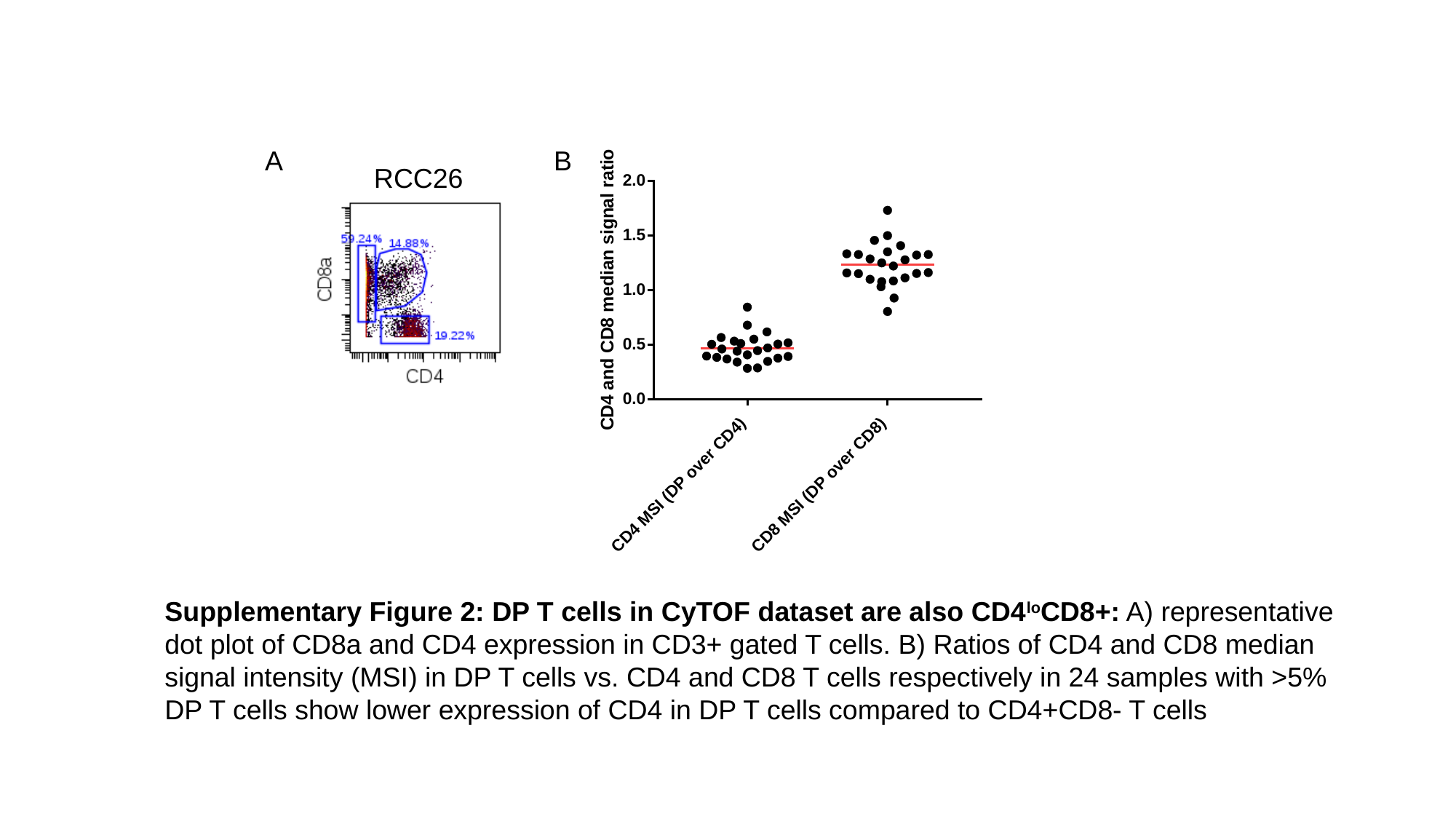

A
B
RCC26
Supplementary Figure 2: DP T cells in CyTOF dataset are also CD4loCD8+: A) representative dot plot of CD8a and CD4 expression in CD3+ gated T cells. B) Ratios of CD4 and CD8 median signal intensity (MSI) in DP T cells vs. CD4 and CD8 T cells respectively in 24 samples with >5% DP T cells show lower expression of CD4 in DP T cells compared to CD4+CD8- T cells

## Slide 3
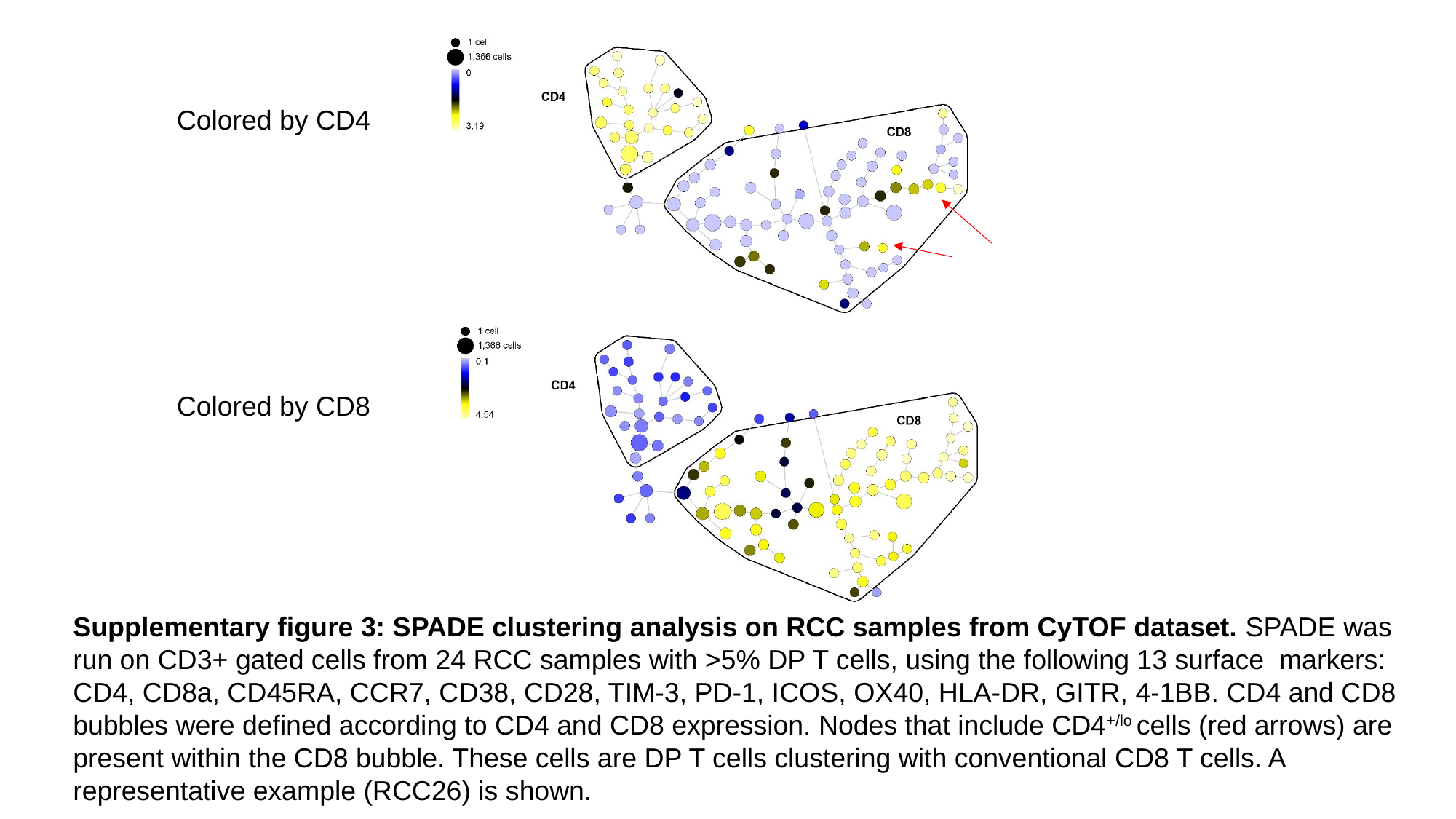

Colored by CD4
Colored by CD8
Supplementary figure 3: SPADE clustering analysis on RCC samples from CyTOF dataset. SPADE was run on CD3+ gated cells from 24 RCC samples with >5% DP T cells, using the following 13 surface markers: CD4, CD8a, CD45RA, CCR7, CD38, CD28, TIM-3, PD-1, ICOS, OX40, HLA-DR, GITR, 4-1BB. CD4 and CD8 bubbles were defined according to CD4 and CD8 expression. Nodes that include CD4+/lo cells (red arrows) are present within the CD8 bubble. These cells are DP T cells clustering with conventional CD8 T cells. A representative example (RCC26) is shown.

## Slide 4
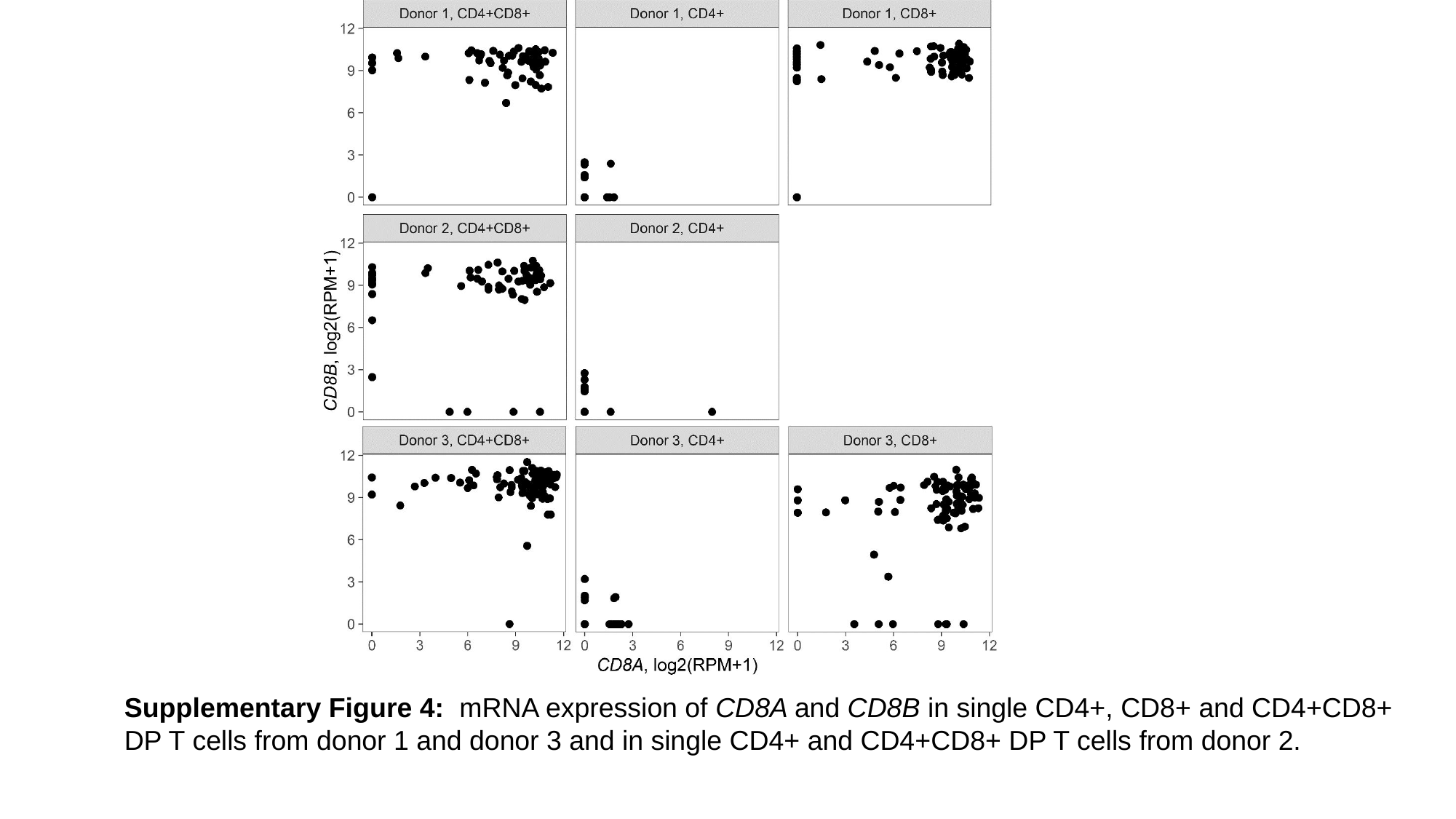

Supplementary Figure 4: mRNA expression of CD8A and CD8B in single CD4+, CD8+ and CD4+CD8+ DP T cells from donor 1 and donor 3 and in single CD4+ and CD4+CD8+ DP T cells from donor 2.

## Slide 5
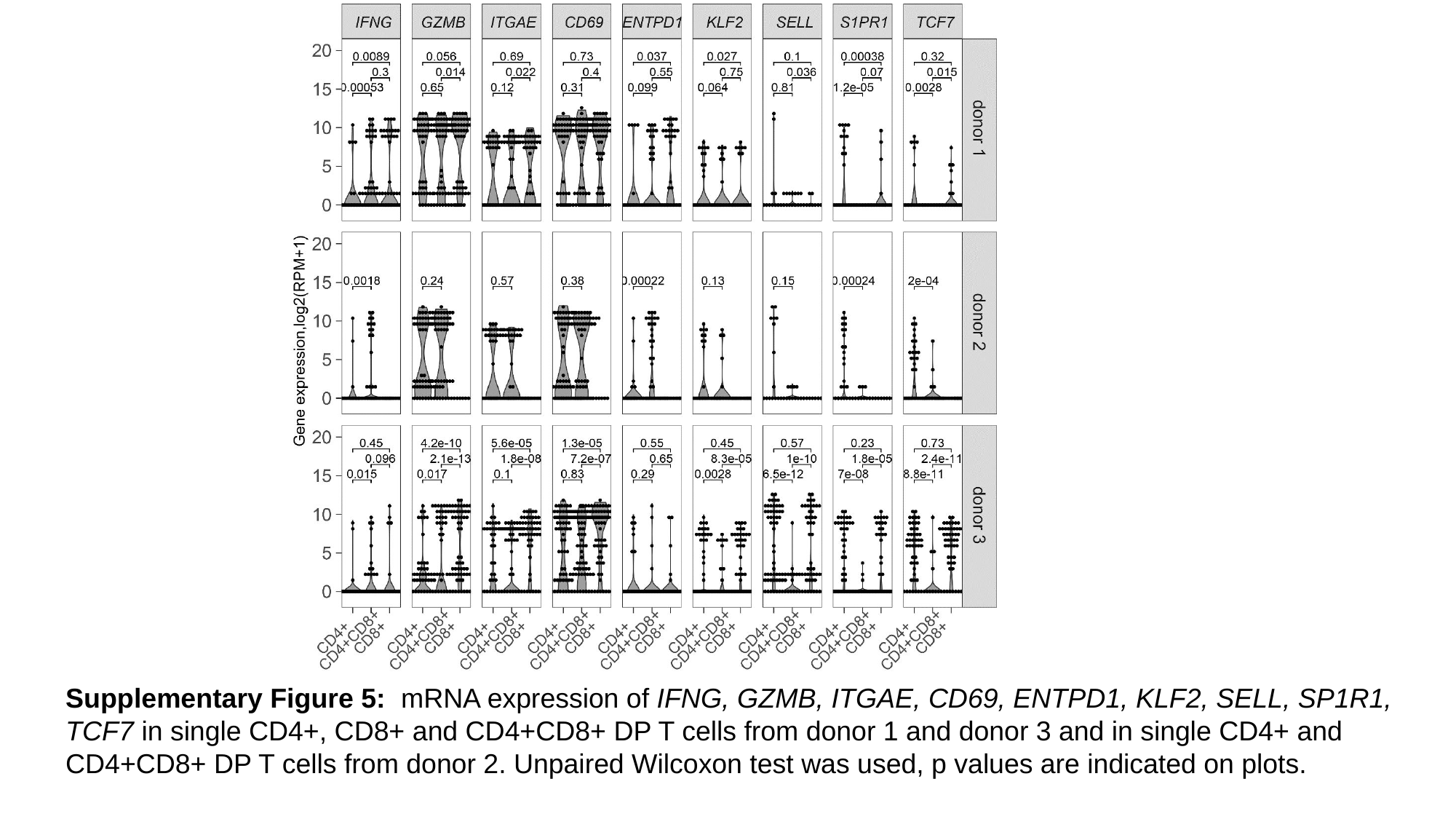

Supplementary Figure 5: mRNA expression of IFNG, GZMB, ITGAE, CD69, ENTPD1, KLF2, SELL, SP1R1, TCF7 in single CD4+, CD8+ and CD4+CD8+ DP T cells from donor 1 and donor 3 and in single CD4+ and CD4+CD8+ DP T cells from donor 2. Unpaired Wilcoxon test was used, p values are indicated on plots.
